# Effects of aversive odour presentation on inhibitory control in the Stroop colour-word interference task

**DOI:** 10.1186/1471-2202-11-131

**Published:** 2010-10-18

**Authors:** Andreas Finkelmeyer, Thilo Kellermann, Daniela Bude, Thomas Nießen, Michael Schwenzer, Klaus Mathiak, Martina Reske

**Affiliations:** 1 Department of Psychiatry and Psychotherapy, RWTH Aachen University, Pauwelsstr. 30, Aachen, Germany; 2 JARA Translational Brain Medicine, Germany; 3 Institute for Neuroscience and Medicine 4, Jülich Forschungszentrum GmbH, Jülich, Germany

## Abstract

**Background:**

Due to the unique neural projections of the olfactory system, odours have the ability to directly influence affective processes. Furthermore, it has been shown that emotional states can influence various non-emotional cognitive tasks, such as memory and planning. However, the link between emotional and cognitive processes is still not fully understood. The present study used the olfactory pathway to induce a negative emotional state in humans to investigate its effect on inhibitory control performance in a standard, single-trial manual Stroop colour-word interference task. An unpleasant (H_2_S) and an emotionally neutral (Eugenol) odorant were presented in two separate experimental runs, both in blocks alternating with ambient air, to 25 healthy volunteers, while they performed the cognitive task.

**Results:**

Presentation of the unpleasant odorant reduced Stroop interference by reducing the reaction times for incongruent stimuli, while the presentation of the neutral odorant had no effect on task performance.

**Conclusions:**

The odour-induced negative emotional state appears to facilitate cognitive processing in the task used in the present study, possibly by increasing the amount of cognitive control that is being exerted. This stands in contrast to other findings that showed impaired cognitive performance under odour-induced negative emotional states, but is consistent with models of mood-congruent processing.

## Background

The olfactory system is an integral part of the paralimbic system; smell can directly modulate emotions and can have profound effects on human cognition and behaviour [1]. Odours may therefore be effectively applied to study the interaction of emotion and cognition. A central aspect of higher cognitive function is the ability to inhibit prepotent responses and irrelevant information. The Stroop task is believed to require such inhibitory processes. Positive emotional states have been shown to increase Stroop interference [2]. In contrast, so far it remains unknown whether negative emotional states can influence performance on the Stroop task in general, and particularly if that holds for emotions that are elicited by smell. The present study seeks to shed light on this aspect of emotion-cognition interaction.

Emotions and emotional states can influence a variety of cognitive functions, and there is increasing consensus that largely overlapping brain networks are responsible for the regulation of both, cognition and emotion [3,4]. However, many aspects of this interaction between emotional and cognitive processing remain unclear (see [5] for a recent review). There are several studies on the role of experimentally induced affective states on cognitive functions such as working memory [6-8] and attention [9]. Negative emotional states induced via video sequences, for instance, impaired the active maintenance of words in a 3-back working memory task, while positive emotional states improved performance, whereas the opposite pattern of results was observed when faces were used as stimuli [7]. This cross-over interaction between affect and stimulus type was also observed in cortical activity in bilateral areas of the lateral prefrontal cortex.

Fewer studies investigated the effect of emotions on inhibitory control functions. Positive emotional states, for instance, have been shown to reduce the ability to inhibit task-irrelevant information [2]. On the other hand, the influence of negative emotional states on inhibitory functions has so far mostly been studied in the context of mood disorders such as depression [10,11], and it is unclear whether these findings are adequate in describing emotional influences on cognition in healthy individuals. It has been shown that negative emotional states can have facilitating effects on executive control in healthy volunteers [12]. However, the different emotional conditions in this study were not under control of the experimenters, and the differences in cognitive functioning may therefore be related to factors other than emotional states. Hence, it remains unclear whether negative emotional states have an effect on inhibitory control. Research on cognitive processing styles, on the other hand, consistently showed that people in a negative emotional state show more focus on detailed information, whereas people in positive emotional states focus more on global information and generally accessible knowledge [e.g. [13-15]]. These findings would predict that negative emotional states increase the ability to exert inhibitory control. To test this hypothesis directly, the goal of the present study was to experimentally induce a negative emotional state and to study its influence on inhibitory control.

To induce the emotional state, the present study used odours, which have been shown to influence mood in a variety of ways [16,17]. Neuroanatomical and neurofunctional findings also show a strong link between olfactory and affective neural processing areas [18-20]. The primary olfactory cortex, for instance, has direct connections to the limbic system, such as the amygdala [21]. Increased activation of the right amygdala has been demonstrated with administration of an aversive odorant and increased activation of the right insula with administration of an appetitive odorant [22]. Both of these brain areas are frequently associated with emotional processing. Odours therefore have the potential to influence affective and cognitive processes in a fairly direct manner, and have been used successfully to investigate their influence on a variety of cognitive processes [1,8,23,24]. Using odours to induce emotional states may also have the advantage that they do not require higher-level cognitive processes (e.g. memory retrieval), and may also be less prone to social expectancy effects in the laboratory context.

Hydrogen sulfide (H_2_S), which has the unpleasant smell of rotten eggs, was used to induce the negative emotional state in 25 healthy participants. We used Eugenol (C_10_H_12_O_2_), the main component in the smell of cloves, as an emotionally neutral odour. Both odorants were administered using a computer-controlled olfactometer, which embedded the respective odorants in a stream of ambient air of constant temperature, humidity and air flow. The odours were presented well above detection threshold. All participants received both odours in two separate experimental runs. Prior to and at the end of each run, participants rated the odours according to intensity, valence and arousal. To reduce potential effects of habituation, the odours were presented alternating with ambient room air, which also served as control conditions.

The influence of the induced emotional state on inhibitory control was measured in the context of the classical Stroop task [[25,26] for a review]. Within each run, performance was compared between blocks of Stroop trials with (H_2_S, Eugenol) and without (ambient air) the olfactory stimulus. In the unpleasant odour run, we expected the Stroop effect to be reduced compared to its non-olfactory control condition, as previous findings demonstrated increased inhibitory control under negative emotional states [12], and decreased control under positive emotional states [2]. Accounts of mood-dependent cognitive processing [13-15] would also suggest increased attention to details of the stimulus in a negative emotional state, and therefore reduced interference from the task-irrelevant stimulus dimension (word meaning). In contrast, the neutral odour should have no influence on the emotional state or on performance in the Stroop task.

## Results

### Odour ratings

The ratings of intensity, valence, and arousal of the two odours were submitted to separate repeated measures analyses of variance (ANOVA) with odour (Eugenol vs. H_2_S) and rating time (pre vs. post run) as within-subject factors and order of odour presentation as between subject factor. Mean ratings are shown in table 1. There was no effect of these factors on rated intensity (all F < 1). As intended, ratings of valence were significantly different between the two odours, F(1,23) = 34.96, p < .001. H_2_S was rated as more unpleasant than Eugenol. Valence ratings did not change from pre to post run, F < 1, nor was there a difference between the two orders of presentation, F < 1. However, there was some indication that the difference in valence ratings between Eugenol and H_2_S may have been influenced by the order in which both odorants were presented (odour-order interaction, F(1,23) = 4.19, p = .052). Participants that were first presented with Eugenol indicated slightly larger differences in valence ratings between both odours, than those participants that first encountered H_2_S (see table 1).

Ratings of arousal were significantly lower for Eugenol than for H_2_S, F(1,23) = 5.63, p = .026. Averaging across odours and presentation order, arousal ratings did not change from pre to post run, F < 1. No other main effect and none of the two-way interactions were statistically significant, all F < 1. The three-way interaction of odour, test time and presentation order was also not statistically significant, F(1,23) = 3.12, p = .091.

**Table 1:** Odour ratings pre and post run

Odour ratings	pre	post	p (pre vs. post)
*H*_*2*_*S*			
Intensity (1 = low, 9 = high)	7.48	7.40	.819
Valence (1 = pleasant, 5 = unpleasant)	4.40	4.20	.134
Arousal (1 = high, 5 = low)	3.84	3.76	.627

*Eugenol*			
Intensity (1 = low, 9 = high)	7.64	7.16	.196
Valence (1 = pleasant, 5 = unpleasant)	3.04	3.24	.233
Arousal (1 = high, 5 = low)	4.32	4.20	.479

The table shows the mean odour ratings of intensity, valence and arousal for the H_2_S and Eugenol runs. Ratings were recorded before and after the individual runs. P-values are shown for a two-sided paired-samples t-test comparing pre- and post-run ratings.

### Stroop performance

Given the low number of erroneous (1.18% of trials, M = 5.68, SD = 4.58) or missing responses (0.57% of trials, M = 2.72, SD = 3.89), accuracy data were not further analyzed. Prior to examining the effects of our experimental manipulations on reaction times (RT), incorrectly answered trials were excluded from the analysis. Post-error slowing was found to be significant, t(24) = 5.10, p < .001. Therefore, reaction times of trials following incorrect or missing responses were also excluded from the analysis (1.59% of the trials).

**Figure 1:**
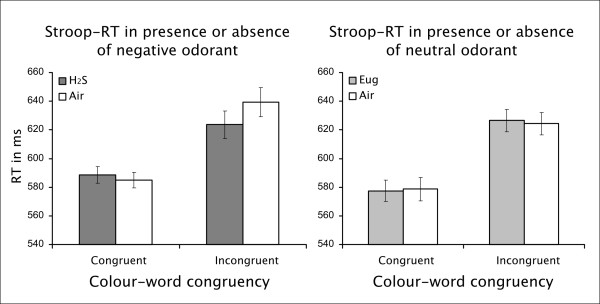
**Stroop Reaction Time Results**. Mean reaction times (and standard error of the mean, after normalisation for subject effects) for conditions of the H_2_S run (left) and Eugenol run (right). In the left panel it is shown that the negative odorant only influenced the reactions time to incongruent stimuli (p = .031), leading to a reduced Stroop effect. Eugenol showed no effect on the measured response times. Note the apparent difference between incongruent trials in the 'air' blocks of both runs, which was not statistically significant (p = .343), however.

Mean reaction times in both odour runs for congruent and incongruent trials in the respective odour and air blocks are shown in Figure 1. To test the central hypothesis of this study, we computed a three-way repeated-measures ANOVA with odour RUN (H_2_S vs. Eugenol), PRESENCE of odour (odour blocks vs. air blocks) and word-colour CONGRUENCY (congruent vs. incongruent trials) as within-subject factors. Results of this ANOVA are shown in table 2. Of the main effects, only word-colour CONGRUENCY had a significant effect on reaction times. None of the two-way interactions were significant. On the other hand, and in line with our hypothesis, the three-way interaction revealed a specific olfactory influence of the unpleasant odour on Stroop interference.

As there was some indication that the ORDER in which participants performed the two runs had a small influence on odour ratings (see ratings analysis above), we included this factor as a between-subjects factor in a second, otherwise identical ANOVA. This resulted in two additional significant interactions (ORDER × RUN, and ORDER × RUN × CONGRUENCY), both of which, however, only indicated practice effects. None of the interaction effects involving both, PRESENCE of odour and ORDER of presentation, were significant. The difference in reaction times between the respective odour and control air blocks was therefore not affected by the order in which the two odours were presented to the participants.

**Table 2:** Results of Analysis of Variance of reaction times

Effect	MS	F	p
*Run *(H_2_S vs. Eugenol)	2699.96	.395	.536
*Presence *(odour vs. air)	398.50	.771	.389
*Congruency *(congruent vs. incongruent)	105684.88	38.327	**<.001**
*Run × Presence*	541.72	.933	.344
*Run × Congruency*	88.39	.091	.766
*Presence × Congruency*	770.48	1.718	.202
*Run × Presence × Congruency*	1603.32	4.412	**.046**

Reaction times were submitted to an odour RUN × odour PRESENCE × Stroop CONGRUENCY repeated-measures ANOVA. All factors were within-subject. Only the main-effect of CONGRUENCY and the three-way interaction effect were significant.

To further characterize the three-way interaction of the first analysis, we computed two separate two-way ANOVAs, one for the H_2_S run and one for the Eugenol run, with odour PRESENCE and word-colour CONGRUENCY as the two remaining factors. The Stroop effect remained significant for both runs as indicated by significantly prolonged RTs for incongruent trials: F(1, 24) = 23.78, p < .001 for the H_2_S run, F(1, 24) = 34.25, p < .001 for the Eugenol run. For the H_2_S run, the main effect of odour PRESENCE was not significant, F(1, 24) = 1.68, p = .208, but there was a significant interaction of PRESENCE and CONGRUENCY in the H_2_S run, F(1, 24) = 6.184, p = .020. As shown in Figure 1, the presence of the negative odorant led to shorter RTs for incongruent trials compared to trials without the negative odorant, t(24) = 2.29, p = .031. RTs for congruent trials were not affected by the presence of the negative odour, t < 1. Similarly, the presence of the neutral odour did not significantly affect performance per se (main effect of odour presence, F < 1) and its interaction with congruency was also not significant, F < 1. Thus, the negative but not the neutral odour significantly shortened reactions times for incongruent trials only. When comparing reaction times between both odour runs for otherwise equivalent conditions (e.g. congruent trials under absence of the odorant), no significant differences could be found (all p's > .230). This indicates that the odorant effects were not large enough to be observed in direct comparisons between the two runs.

## Discussion

In the present experiment we investigated the influence of odours with different emotional valence on the performance in a standard colour-naming Stroop task. Our reaction time analysis showed a reduction of the Stroop effect specifically during the presence of an odorant with negative valence and high arousal (H_2_S) compared to a condition in which it was absent. This reduction was the result of changes in reaction times for incongruent trials only. It appears that the negative emotional state induced by the odour resulted in a reduction of cognitive interference for incongruent stimuli, because it was easier to inhibit the task irrelevant information (word meaning) in the stimulus. This finding of reduced Stroop interference due to negative olfactory stimulation is compatible with the idea of a more analytic or focused style of processing under a negative emotional state [13,14]. The task of naming the print colour (as opposed to reading the word) would be enhanced by such a processing style and thereby lead to faster reaction times for the incongruent stimuli.

An alternative interpretation of the observed effects in the H_2_S run is based on the fact that, in cross-run comparisons, RTs for incongruent trials numerically differed the most in the air conditions (not the odour conditions), so that differences between the H_2_S and Eugenol runs were primarily due to differences in the conditions in which these odorants were not present. This interpretation would mean that we observed an increase in RTs for incongruent trials in the absence of the negative odour instead of a decrease in RTs for incongruent trials in its presence. This interpretation may at first seem counterintuitive, as it would mean that conditions that are chemically identical (i.e. odour absent conditions of both runs) resulted in different responses, while conditions that differ chemically and in their psychological ratings (odour present conditions) did not affect performance directly. Given that significant differences between air and odour conditions were found only in the H_2_S run, it is clear that this odorant, and the negative emotional state it induced, must in some way be responsible for these differences. One possible explanation is that the negative odour primarily had an indirect influence, such that participants experienced a temporary positive emotion (e.g. 'relief') when stimulation with the negative olfactory stimulus was temporarily turned off during the H_2_S run. This positive emotion may then have resulted in reduced inhibition and thus increased Stroop interference, an interpretation that would be consistent with previous findings on Stroop performance [2]. However, since no positive odour condition was tested in the present experiment, and because post-hoc comparisons between the negative and neutral odour runs did not show significant differences, it remains unclear from our results whether the effects of the negative odour on performance were direct or indirect.

In contrast to the unpleasant odour, the presentation of Eugenol had no consistent effect on reaction times. Neither congruent nor incongruent trials were processed differently under the presence of Eugenol than under control air. Intensity judgments did not differ between Eugenol and H_2_S, and could therefore not be the cause for the observed difference between the two odorants. The medium ratings of pleasantness and low ratings of arousal of Eugenol suggest that its presentation had no or little effect on the emotional state of the participants. On the other hand, the presentation of H_2_S seemed to induce a strong negative emotional state in the volunteers, as reflected in higher levels of perceived unpleasantness and arousal. The observed effects during the H_2_S run can therefore not be explained by a general attention mechanism that is recruited by the mere presence of an odorant. The observed modulation of the Stroop effect must therefore be related to the emotional state that was induced by the unpleasant odorant. Furthermore, while it is possible that a presentation of a pleasant odorant would result directly in an enlargement of the Stroop effect, the control odour that was used in our study did not appear to induce a positive emotional state and also failed to modulate the Stroop effect.

The observed modulation of Stroop interference by the negatively valenced H_2_S parallels findings on the recruitment of cognitive control in the Stroop task [27-29]. In analyses of trial-by-trial adjustments of performance, these studies showed that the Stroop effect is reduced following incongruent trials. The authors argue that the response conflict of the incongruent trials results in recruitment of control mechanisms that reduce the processing time for the following trial, if it is also an incongruent trial, and increases processing time for the following trial, if it is a congruent trial. Results of an fMRI study [29] revealed left middle and superior frontal gyrus activation for trials with increased cognitive control. This finding is consistent with the notion that negative emotional states increase withdrawal related activity in the left prefrontal cortex, whereas positive emotional states lead to a decrease in activity in the left prefrontal cortex [7]. This structure has also been implicated in the behavioural adjustment following response conflict [28]. The observed modulation of Stroop interference via negative olfactory stimulation in the present study could therefore be due to a mechanism that is closely related to the recruitment of cognitive control during post-conflict adjustment. If the negative odours modulated activity in the left prefrontal cortex, it is possible that it hereby also modulated the ability to recruit cognitive control mechanisms during performance of the Stroop task. Further fMRI studies could shed light on this hypothesis.

Previous research on the effect of negative olfactory stimulation on cognitive functioning also found detrimental effects on performance in a working memory task, though there were large inter-individual differences in the degree to which performance was impaired by a negative odour [8,30,31]. It appears that, while the active maintenance of information in working memory may be impaired by negative olfactory stimulation, the inhibition of dominant or automatic response tendencies in the Stroop task might be enhanced by it. This differential effect of negative olfactory stimulation on cognitive function may be related to general task difficulty. Performance in a rather simple task like the Stroop task might benefit from the increased arousal following the onset of the negative odorant, while a more complex working memory task is impaired by the additional arousal. Such an interpretation would be compatible with the well-known non-linear relationship between arousal and performance first described by Yerkes and Dodson [32]. Further studies are needed to address the possible differential roles of valence and arousal on performance and their interaction with task difficulty.

Several limitations of the current study should be mentioned. As stated above, we did not use a pleasant, positive odour, which would have allowed the testing of several alternative explanations for the observed effects. Second, the unpleasant and the neutral odour did not only differ in their valence, but also in the degree of arousal they caused. Thus, the effect of H_2_S on Stroop performance may also be related to increased arousal and not its unpleasantness alone. Lastly, we did not assess the emotional state of the participants directly, but rather asked them to judge how they perceived the olfactory stimuli in terms of their intensity, valence and arousal. It is possible that the affective state of the participants differed from their judgements of the odours as they may have engaged in processes that regulated their emotional state.

## Conclusions

We studied the influence of a negative and a control odour on the Stroop effect. In this paradigm, inhibitory control was modulated by an olfactory induced emotional state, either by directly reducing Stroop interference during presence of the negative olfactory stimulus, or by indirectly increasing Stroop interference during the temporary interruption of negative odour presentation. The data argue for a partial overlap of emotional processing with cognitive control mechanisms, but extensions to positive valence, designs focusing directly on cognitive control, and neuroimaging could help to shed light on the exact nature of this emotion-cognition interaction. The use of olfactory stimulation to induce emotions provides an experimental paradigm that can modulate emotions controlled and rapidly. The involved paralimbic networks may give a better insight into the neural networks underlying emotions and their effects on cognitive process.

## Methods

### Participants

Twenty five healthy volunteers (7 female) between the ages of 22 and 58 years (M = 32.1, SD = 10.3) participated in the study. All participants were native German speakers. Participants were tested for olfactory function using the identification subtest of the Sniffin' Sticks Test [33], a multiple choice screening test of olfactory function. All participants identified at least 10 of the 12 tested smells correctly meaning that no participant had to be excluded due to basic olfactory deficits. Participants had a mean education of 13.2 years (SD = 2.5). Verbal crystalline intelligence was assessed for each participant using the MWT-B [34], a forced choice word recognition test. IQ scores ranged from 95 to 136, with a mean score of 115.4 (SD = 12.8). Participants received €10 for their participation. The research was approved by the research ethics committee of the School of Medicine, RWTH Aachen University (reference number EK 073/05), and all participants gave written informed consent prior to participating in the experiment.

### Apparatus and Stimuli

The visual stimuli were presented via a laptop computer with a 15 inch (38.1 cm) screen. It was placed in front of the participants at a distance of 60 cm. Visual stimulus presentation and data collection were performed using the software package Presentation (Neurobehavioral Systems Inc., San Francisco, CA). Words naming the colours red and green were presented in the centre of the screen in upper case letters ("ROT" red, "GRÜN" green). They were displayed on a black background in either red or green font. The words had a height of 1.8 cm and a width of 5.0 cm (red) and 7.2 cm (green). Each stimulus was presented for 1500 ms, with an interstimulus interval of 1500 ms, during which a white fixation cross was presented in the centre of the screen. Participants indicated the colour of each word by pressing one of two keys on the laptop's keyboard with the index and middle finger of their right hand (index finger for words presented in red, middle finger for words presented in green). This colour-to-key mapping was identical for all participants.

Olfactory stimulation was performed using an olfactometer (Burghart Medizintechnik, OM-4b), which allows the controlled delivery of odorants in a stream of humidified air with constant gas flow (8 l/min) and temperature (37° Celsius). Two different pure olfactory odorants without trigeminal component were presented. H_2_S solved in nitrogen (20 parts per million) was used as the negative olfactory stimulus. The nitrogen-H_2_S gas mix was further diluted with ambient air at a ratio of 7 parts gas mix (3.5 l/min) to 9 parts air (4.5 l/min). Eugenol solved in propylene glycol (C_3_H_8_O_2_; 6.2 per 100 ml) was used as the other olfactory stimulus [35]. This preparation is liquid at room temperature and was diffused using one of the olfactometer's built-in diffusers. In general, Eugenol has a pleasant spicy smell and should therefore not elicit strong negative emotions. Nevertheless the valence ratings for Eugenol can vary and even show trends towards the negative direction [36]. Individual ratings of odorant qualities were conducted in all participants. The respective odorant-air mix was delivered to the right nostril of the participants, intermitted with ambient air without the addition of an odorant (see below).

### Procedure

All participants completed two runs of trials, each using one of the two odorant stimuli. The order of the two odour runs was counterbalanced across participants. The influence of the odorants on the affective state of the participants was assessed through ratings of intensity, pleasantness, and level of arousal associated with the odours. Prior to the beginning of each run, participants were asked to give these ratings after having received a short 2 second presentation of the odorant used during that run. Intensity ratings ranged from 1 (no perception of the odour) to 10 (very intense odour smell). Valence and arousal ratings were given on a scale from 1 to 5, with 1 representing 'very pleasant' (valence) or 'very nervous' (arousal), and 5 representing 'very unpleasant', or 'very calm'. Participants performed these judgments again at the end of each run. While this method does not directly assess emotional state of the participants during the cognitive task, it avoids possible demand characteristics of other mood assessment techniques and may also be more sensitive to differences between the two odorants.

Participants performed 240 trials of the Stroop task per run. The total length of each run was 12 minutes. During each run, the olfactometer switched between (non-odorous) ambient air and the current odorant-air mix every 8 trials (i.e. every 24 seconds) thereby creating 15 blocks of 'air' trials and 15 blocks of 'odour' trials. Runs always started with an air block and ended with an odour block. One of two pseudo-randomized orders of Stroop stimuli was used that insured an equal number of congruent and incongruent Stroop stimuli among 'air' trials and 'odour' trials.

### Statistical Analysis

Data were analysed using repeated-measures Analyses Of Variance (ANOVA) using the SPSS statistics software package (SPSS Inc., Chicago, IL). The factors experimental RUN, odour PRESENCE, and word-colour CONGRUENCY were included in these analyses. Post-hoc comparisons were performed using matched-samples t-tests.

## Authors' contributions

MR, KM and TK conceived of the study and participated in its design. DB, MS and TN participated in the collection of the data. AF carried out the data analysis and interpretation, and drafted the manuscript. All authors read, critically revised and approved the final manuscript.
